# Making Self-Management Mobile Health Apps Accessible to People With Disabilities: Qualitative Single-Subject Study

**DOI:** 10.2196/15060

**Published:** 2020-01-03

**Authors:** Leming Zhou, Andi Saptono, I Made Agus Setiawan, Bambang Parmanto

**Affiliations:** 1 Department of Health Information Management University of Pittsburgh Pittsburgh, PA United States; 2 Department of Computer Science Udayana University Badung, Bali Indonesia

**Keywords:** mobile app, self-management, accessibility, personalization

## Abstract

**Background:**

Over the past decade, a large number of mobile health (mHealth) apps have been created to help individuals to better manage their own health. However, very few of these mHealth apps were specifically designed for people with disabilities, and only a few of them have been assessed for accessibility for people with disabilities. As a result, people with disabilities have difficulties using many of these mHealth apps.

**Objective:**

The objective of this study was to identify an approach that can be generally applied to improve the accessibility of mHealth apps.

**Methods:**

We recruited 5 study participants with a primary diagnosis of cerebral palsy or spinal cord injury. All the participants had fine motor impairment or lack of dexterity, and hence, they had difficulties using some mHealth apps. These 5 study participants were first asked to use multiple modules in the client app of a novel mHealth system (iMHere 2.0), during which their performance was observed. Interviews were conducted post use to collect study participants’ desired accessibility features. These accessibility features were then implemented into the iMHere 2.0 client app as customizable options. The 5 participants were asked to use the same modules in the app again, and their performance was compared with that in the first round. A brief interview and a questionnaire were then performed at the end of the study to collect the 5 participants’ comments and impression of the iMHere 2.0 app in general and of the customizable accessibility features.

**Results:**

Study results indicate that the study participants on their first use of the iMHere 2.0 client app experienced various levels of difficulty consistent with the severity of their lack of dexterity. Their performance was improved after their desired accessibility features were added into the app, and they liked the customizable accessibility features. These participants also expressed an interest in using this mHealth system for their health self-management tasks.

**Conclusions:**

The accessibility features identified in this study improved the accessibility of the mHealth app for people with dexterity issues. Our approach for improving mHealth app accessibility may also be applied to other mHealth apps to make those apps accessible to people with disabilities.

## Introduction

### Background

Currently, approximately 650 million people in the world live with a disability. In the United States alone, there are 61.4 million adults living with a disability, which is 25.7% of the US adult population [1]. The aging population in the United States has resulted in a steady increase in this percentage in the past several years because prevalence of any disability is higher among older age groups [2]. If there is no specific support provided to these people with disabilities, they will have serious difficulty taking care of themselves.

Mobile health (mHealth) apps offer one way to provide desired support to people with disabilities so that they can perform some health self-management tasks and achieve a certain level of independence. As of December 2017, 325,000 mHealth apps had been created [3]. The general purpose of patient-oriented mHealth apps has been to assist patients to manage their own health or receive desired health care services from their health care providers when a face-to-face meeting is not feasible [4-11]. A number of studies have been performed to evaluate the usefulness and effectiveness of these mHealth apps [12-19], and a number of apps have proven to be useful and effective in maintaining or improving people’s health [12-20].

Only a small number of mHealth apps, though, have been specifically designed for people with disabilities, and an even smaller number of apps have undergone accessibility evaluation with people with disabilities [21-26]. In other words, although an enormous number of mHealth apps have appeared on the market, only a very limited number of them may be used by people with disabilities, thereby increasing health care service disparities between people with disabilities and the general population [27]. Therefore, more mHealth apps designed for people with disabilities that include features for self-management [17,28] of health care and that are highly accessible [29-31] are highly desirable.

People with different disabilities have different needs in terms of accessibility. For example, visually impaired people need magnifiers, audio alerts, or screen readers to access the information from a mobile app; people with hearing impairment may need flashing lights, vibration capability, or caption services to receive information from a mobile app. This difference in needs means it is important to work closely with target users when designing accessibility features in mHealth apps.

### Previous Work

In the past two decades, although many accessibility studies have been conducted, many were related to Web accessibility on computers. Only a small number of accessibility studies investigating mobile app accessibility have been conducted, and only a few of these studies focused on mHealth app accessibility.

Among the studies on mobile app accessibility, authors have evaluated various approaches for improving the accessibility of their apps, for instance, creating different user interfaces for people with different types of disabilities such as visual [32,33], hearing, physical, and cognitive impairment [34]; designing user interfaces and information and communication technology systems for older adults [35,36]; building a customized mobile app for people with cerebral palsy (CP) [37]; and generating accessibility toolkits [38].

Several years ago, the first version of a novel mHealth system named iMHere 1.0 was created to help patients to manage chronic conditions [11]. The accessibility of the iMHere 1.0 app was evaluated in patients with dexterity impairment [29-31]. In iMHere 1.0, a number of accessibility features were implemented, for instance, simplified steps for entering information about medications, color-coded themes, a customizable app list, adjustable text and button size, and color-coded contents [30,31].

Recently, an updated version of the mHealth system, iMHere 2.0, was created to support a variety of self-management tasks for people with disabilities [28,39]. Moreover, a component of this updated mHealth system is an app used by people with disabilities (referred to as *client app* in the following descriptions). As this client app was implemented with cross-platform packages, it can run on all 3 major platforms: iOS, Android, and Windows phone systems. In the iMHere 2.0 client app, there are 12 app modules for different self-management tasks, such as medication management, mood self-assessment, and minor skin problem reporting. A usability study with 81 study participants from the general population was performed on the iMHere 2.0 client app, and the results indicated that the app had high usability for people in the general population [39].

### Objectives

The objective of this project was to evaluate and improve the accessibility of the mobile client app in the iMHere 2.0 system to support self-management and personalized care. Our ultimate goal was to identify an approach for making mHealth apps accessible to people with disabilities.

## Methods

### Study Participant Recruitment

The study protocol (PRO18020101) was approved by the institutional review board (IRB) office at the University of Pittsburgh. The study participants were recruited via referral. The selection criteria were being a native English speaker aged between 18 and 65 years with CP, spina bifida (SB), or spinal cord injury (SCI) and with a disability in fine motor skills. A phone screening was conducted with each referred potential study participant to verify the information we had obtained from clinicians, such as the potential participant’s willingness to participate in the study, their primary diagnosis, and their impairment in fine motor skills.

### Study Procedure

Before the beginning of the study, each study participant was given sufficient time to read the IRB-approved consent form carefully and to sign the form if the contents were acceptable. The study participation was completely voluntary, and the participants could leave the study at any time. After the consent form was signed, a general introduction to the study purpose and procedure was provided to these study participants.

Each study participant was then required to take a few standard tests to evaluate their vision, cognitive level, and dexterity. The Snellen eye chart was used for vision assessment, the Montreal Cognitive Assessment for cognitive level assessment, and the Purdue Pegboard Test (Model 32020A) for dexterity assessment.

Dexterity impairment level was determined based on the number of pins picked up from a shallow cup and plugged into holes on a board in 30 seconds, using his or her left and right hand, respectively. The smaller the values, the more severe the dexterity impairment. If the value was 0, it meant the study participant was not able to pick up any pin or plug it into a hole on the board.

After these standard tests, an interview was conducted to obtain a better picture of the situation of each participant, for instance, their disability, primary diagnosis, type of wheelchair used, difficulties in daily life, mobile devices used frequently, number of years using mobile devices, difficulties using mobile devices, and assistive technologies used in the past.

After the interview, a demonstration of 5 modules (skincare, mood, education, nutrition, and exercise) in the client app of the iMHere 2.0 system was provided to the study participants [39].

The study participants were then asked to use these app modules one by one and finish several tasks, one in each module. The following is a list of tasks they were required to finish:

- Reporting a skincare case in the skincare module.
- Performing a self-assessment on mood in the mood module.
- Adding records in the exercise module.
- Adding records in the nutrition module.
- Reading a few sections of education materials in the education module.

Study participants were also encouraged to use other modules offered in the app such as MyMeds for medication management and PHR for personal health records management. Their performance on all these modules was observed and noted, for instance, the number of attempts they needed to successfully click an indicated user interface component such as a button, whether they accidentally clicked a nearby button, and whether they had difficulties reaching the indicated button. Besides typical buttons, the indicated interface components in this study also included radio buttons, pictures, check boxes, icons in the app, text boxes, hyperlinks, keys on the soft keyboard, and arrows (eg, left, right, up, down, and back to the previous page).

An iPhone 6 plus, a 9.7-inch iPad Air 2, and a 9.7-inch Samsung Android tablet provided by the study team were used at this stage. Each study participant was only *required* to use one of these 3 devices according to their situation and the type of devices and the mobile operating systems they were familiar with. This arrangement was to remove any accessibility issues that could be introduced by having to use an unfamiliar mobile device or operating system. They were allowed to use the app on other devices as well if they chose to do so.

The study participants were interviewed to collect their feedback on the accessibility of the iMHere 2.0 client app and their desired accessibility features in the mHealth app. The accessibility features that the study participants expressed a desire to have were then summarized, analyzed, selected, designed, and implemented in the app.

As different people with disabilities have different requirements for accessibility, multiple types of accessibility features were made available and *customizable* in the app. People with disabilities then had the ability to customize the app interface according to their own needs.

During the accessibility feature selection stage, we evaluated each requested accessibility feature by asking 2 questions: (1) Is this request a need or preference? and (2) Will this feature improve the accessibility of the app for *this population*? If the feature was only a preference, not a need, it did not have high priority on the feature implementation list. If the feature could not improve the accessibility of the app for this particular population, we did not add it into the app.

It took us more than 3 months to discuss, design, and implement these accessibility features. After these features were added into the iMHere 2.0 client app, the 5 study participants were invited to use the same modules in the iMHere 2.0 app again and finish the same tasks with and without the newly implemented accessibility features. Their performance was again observed and noted.

After the study participants finished all the tasks, they were interviewed to determine whether the accessibility of the app had improved by orally giving a rating to the following 4 statements on a scale ranging from 1, strongly agree, to 7, strongly disagree.

*These accessibility features make it easier for me to:*

1. click the desired buttons.

2. make selection in a list of options in the app.

3. understand the content in the app.

4. navigate different pages in the app.

All 5 participants were also required to respond to the 10 usability statements in the System Usability Scale (SUS) via the Web-based Qualtrics system [40] to express their overall impression of the usability of the iMHere 2.0 client app. A brief and informal interview was performed to verify their answers on the questionnaire.

For study participants who used a power wheelchair and had a joystick on the wheelchair, a test was performed to determine whether they could use the joystick on their wheelchair to perform navigation and item selection in the iMHere 2.0 client app on the mobile devices.

All the data collected in these steps were summarized and analyzed to draw conclusions.

### iMHere 2.0 Client App

The iMHere 2.0 client app was designed to support patients’ self-care tasks, send data to clinicians, and allow patients to receive personalized regimens from their clinicians [39]. This app has 12 modules in total, 5 of which were used in this study: skincare, mood, exercise, nutrition, and education. The skincare module can be used to report minor skin problems by indicating the wound site, taking a picture, and sending it to the provider along with answers to a few questions about the wound situation. The mood module is used to perform mood self-assessment by answering the Patient Health Questionnaire-2 (PHQ-2) for depression screening and the Generalized Anxiety Disorder-2 (GAD-2) for anxiety assessment [41,42]. The exercise module can be used to track physical activities each day by having the user select the type of activities completed from an activity icon library and indicate the duration of each activity. The nutrition module is used to track types of food and drink consumed by the app user each day. The education module includes information on various topics related to the health of people with disabilities, such as background information on CP and SB. Each of these modules has unique user interface components.

Moreover, 2 other modules were made available during the study: MyMeds for medication management and PHR for personal health records management. They were not required in the study because they had useful features for users but did not include unique user interface components. These 2 modules were provided so that these study participants could explore the app further if they wanted.

**Figure 1:**
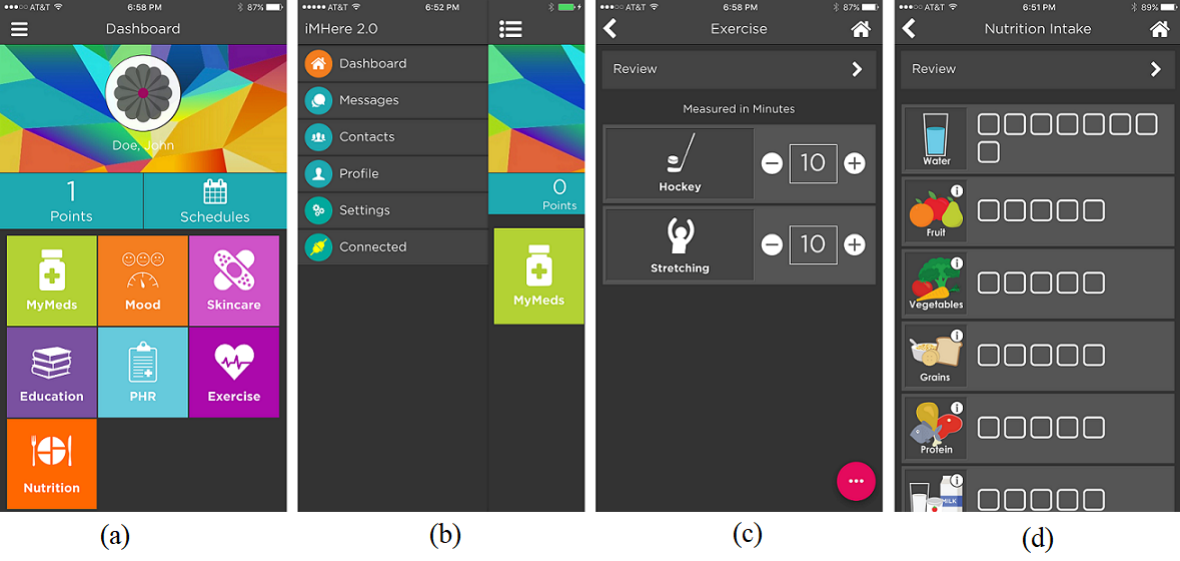
Screenshots of the iMHere 2.0 (a) dashboard, (b) menu buttons, (c) exercise module, and (d) nutrition module.

As shown in Figure 1, the home screen of iMHere 2.0 consists of 2 main areas: one is the dashboard with a user name, reward points, schedules, and a list of modules (see the first diagram in Figure 1), and the other is a list of menu buttons, which appears after the list icon at the top-left corner is clicked (see the second diagram in Figure 1). One of the menu buttons is a link to the system settings page (not shown). All the modules shown in the dashboard can be launched by clicking on their icon. Once the icon of a module is clicked, the module takes the user to the corresponding app’s main screen (the third and fourth diagrams in Figure 1 are for the main screen of the exercise and nutrition modules).

## Results

### Demographic and Basic Information

In this study, 5 participants were recruited from the Greater Pittsburgh area via referral. The ages of the 5 study participants were 18, 28, 33, 35, and 41 years; their average age was 31 (SD 8.63) years. All of them (5/5, 100%) were white Americans. The average number of years using smart devices was 8.8 (SD 1.10). Other demographic information is summarized in Table 1. Their standard test results, hand preference, and difficulty performing daily activities are shown in Table 2.

**Table 1:** Demographic characteristics of the study participants (N=5).

Characteristics		Value, n (%)
**Gender**		
	Male	2 (40)
	Female	3 (60)
**Education**		
	High school	1 (20)
	Bachelor’s degree	3 (60)
	Master’s degree	1 (20)
**Employment**		
	Employed	2 (40)
	Not employed	3 (60)
**Marital status**		
	Single	4 (80)
	Married	1 (20)
**Primary diagnosis**		
	Cerebral palsy	4 (80)
	Spinal cord injury	1 (20)
**Primary mobile device**		
	iPad	1 (20)
	Android phone	2 (40)
	iPhone	2 (40)
**Wheelchair**		
	Power wheelchair with Bluetooth connection	3 (60)
	Power wheelchair without Bluetooth connection	1 (20)
	Manual wheelchair	1 (20)

**Table 2:** Standard test results, hand preference, and difficulty performing daily activities.

Prestudy evaluations			Value, n (%)
**Standard test results**			
	**Vision (both eyes)**		
		20/20	4 (80)
		20/25	1 (20)
	**Cognitive level**		
		Normal	2 (40)
		Below average	3 (60)
	**Dexterity (left/right)**		
		0/0	3 (60)
		10/3	1 (20)
		0/5	1 (20)
**Hand preference**			
	Right		3 (60)
	Left		2 (40)
**Daily activity**			
	**Feeding oneself**		
		Yes	1 (20)
		Yes with special tools	1 (20)
		No	3 (60)
	**Using restroom independently**		
		Yes	0 (0)
		No	5 (100)
	**Needing reminder to take medications**		
		Yes	1 (20)
		No	4 (80)

### Subject-Specific Information

Participant 1 was an 18-year-old female high school student. Her primary diagnosis was CP. She also had experienced seizures and had type 1 diabetes. Her dexterity impairment was severe. The participant mainly used an iPad at home and in school. She strongly depended on her iPad to communicate with others and finish her schoolwork. Her spoken language could be understood by her family members, teachers, and friends but was difficult to understand for others. The joystick on her power wheelchair could be paired with other devices (such as a music player) via Bluetooth. She preferred to use her iPad and the soft keyboard on it to finish various tasks. She could use her fingers and the joystick on her wheelchair to finish those tasks. She could use her wheelchair to move around, but she needed others (eg, family members and school staff) to help her to finish many daily activities such as having a meal, taking a shower, and using a restroom.

Participant 2 was a 41-year-old male. He had a part-time job. His primary diagnosis was SCI. He used a power wheelchair. His spoken language was easy to understand for everyone. He commonly used a desktop computer and an Android phone. He did not own a tablet. He could use mobile apps on his Android phone. His power wheelchair had Bluetooth, but he had never tried to use it to pair with other devices. In most cases, he needed a dedicated caregiver to help him to finish daily activities. Sometimes, he used specially designed tools to feed himself. His dexterity impairment was moderately severe. He could use the back of his fingers to make selections on a mobile device. His arms had a very limited range of movement; therefore, he had significant difficulty using mobile devices with a bigger screen, such as a 10-inch iPad.

Participant 3 was a 35-year-old female. She had a bachelor’s degree and a full-time job. Her primary diagnosis was CP, and she used a power wheelchair. Her dexterity impairment was moderately severe. Her wheelchair did not have Bluetooth. She was able to use a desktop computer, laptop, iPhone, iPad mini, and iPad. Her spoken language was sometimes difficult to understand.

Participant 4 was a 33-year-old male. He had a bachelor’s degree and was looking for a job. His primary diagnosis was CP, and he had Parkinson disease as well. He had both a manual wheelchair and a power wheelchair. He used the manual wheelchair at home. He used an Android cell phone and could use apps on the phone. His dexterity impairment was mild.

Participant 5 was a 28-year-old female. She had a master’s degree and had a full-time job. Her primary diagnosis was CP. She used a power wheelchair with Bluetooth. She could use mobile apps on her iPhone and iPad. Her dexterity impairment was moderate.

All the study participants took multiple types of medications daily. Moreover, 4 of them (80%) needed other people’s help to take the medications on time. They all had mobile devices and could use some apps on the mobile devices. They all had used smart mobile devices for a number of years. None of them could use the restroom independently. Most of them (4/5, 80%) could only use 1 hand to operate mobile devices.

### Performance on iMHere 2.0 Without Accessibility Features

Overall, all the participants were able to follow the investigator’s instructions, click the indicated buttons and options, take pictures, find the desired pages, and enter the information with different levels and types of difficulties. It was relatively easier for them to make selections at the left or right edge of a tablet or phone. It was difficult for them to perform selections when buttons or options were in the middle of a tablet or when they were only available on the left- or right-hand side instead of both sides, as most of them (4/5, 80%) could only use one hand to operate the mobile device.

Participant 1 had difficulty zooming in and out on a tablet using one hand or pinpointing a specific location for button clicking or typing. She made multiple attempts to finish tasks such as clicking a button, making selections of investigator-indicated options, or entering a word. She strongly depended on the word prediction feature of the soft keyboard to finish her word typing. If buttons were big and far away from each other, it was easier for her to make the selection. It was easy for her to swipe to move to different pages on the tablet.

Participant 2 had difficulty using the app on a 9.7-inch tablet as his arm had only a very small range of movement, and he could only use the back of his fingers of his left hand to make selections. His performance was much better when he used the app on the iPhone. On the phone, he was able to make the desired selections and type words into the app. He made multiple attempts before achieving success when a button only occupied a small area. He made a few mistakes when typing words.

Participant 3 could easily make selections when the buttons or options could be accessed from the left-hand side. Selections were especially easy for her when she was working on a large tablet. When she entered text on the app, she made many mistakes but could enter the correct text eventually. It was relatively difficult for her to select buttons located on the right-hand side or in the middle of the tablet screen, requiring multiple attempts. When she worked on the iPhone, the situation was better as she was able to access almost all the buttons and options from the left-hand side of the phone.

Participant 4 did not have any major difficulty finishing all the given tasks, whether on the iPhone or the tablets.

Participant 5 could make selections on both the iPhone and iPad using her right hand. She had difficulty when selecting some options, for instance, the pop-up menu in the skincare module. Sometimes, she experienced difficulty entering text. It was also difficult for her to select buttons located on the left-hand side or in the middle of the tablet screen.

All participants briefly explored the MyMeds and PHR modules after they finished the required tasks in the 5 modules. They all indicated that the availability of these 2 modules was very useful for their health management as the MyMeds module can remind them to take medications on time every day if needed, and the PHR module can store all their essential personal health records in one place.

### Feedback and Desired Accessibility Features

The following is a summary of the feedback and desired accessibility features from these 5 study participants.

#### Font Size and Style

Although all the study participants could read the text contents of the app, multiple participants (3/5, 60%) expressed the desire for larger font sizes and bold style in certain cases. Some also expressed a desire for font size and style of text on the app to be adjustable according to their needs.

#### Spacing

Most of them (4/5, 80%) did not like dense reading material or buttons close to each other. They expressed a desire for the spacing between lines and buttons to be adjustable.

#### Button and Selection Option Arrangement

Most of the participants (4/5, 80%) had difficulty clicking buttons located in the middle of the screen, especially on a large tablet. Therefore, they preferred that buttons could be moved to the left edge and right edge, with multiple buttons aligned vertically. This also applied to options to questions. Instead of only allowing for radio button or check box selection, they desired that the option selection should be done anywhere from left to right as a long button.

#### Color and Contrast

All the participants liked the use of different colors for different pages in different app modules. At the same time, they pointed out that some colors were too light and that sometimes the contrast between the background and the text was not high enough. They felt that a simple white background and dark text or vice versa would be better. They also expressed a preference for the color of a button or option to change in acknowledgment of a selection having been made. Without that, they reported that they might keep trying and become frustrated because they thought they had not successfully clicked the desired button or option.

#### Alternative Approach for Data Input

Most of these participants (4/5, 80%) experienced some level of difficulty typing words using the soft keyboard on the app. These participants typically had to try multiple times to enter one word. It was easier for them to select from among given options. Therefore, they expressed a desire that the app provides options for them to select instead of asking them to type in words to describe their situation, for instance, providing a picture or list of body parts to complete minor skin issue reporting in the skincare module.

#### Page Navigation

All the study participants noticed that some pages in the education module were longer than one screen and that there was no indicator of that. They reported that it would be better to have a scroll bar to indicate this fact. An alternative approach would be to split the page longer than one screen into multiple pages as all these participants were able to perform a swipe between pages easily. They also expressed that left and right arrows on a page were needed so that the page navigation could be performed using other approaches or tools than fingers, such as a paired joystick. Similarly, up and down arrows were requested as well for when the content was longer than one page on the mobile device screen.

#### Handedness

Most of these study participants (4/5, 80%) could only use one hand to operate the mobile devices, and it was easier for them to make selections on the side convenient for their hands. Therefore, they expressed the desire for an option for users to choose the handedness of the buttons and options.

#### Multimedia Contents

Some study participants (3/5, 60%) expressed a desire for screen reading, pictures, audio, and video content for the education materials.

They also desired to have *individual* accessibility setting changes (font size, font style, button size, spacing, and handedness) instead of choosing from a few built-in accessibility templates.

### Accessibility Features Implemented

The iMHere 2.0 client app was modified to include the features participants had expressed a desire for. First, easily adjustable font sizes, font styles, line spacing, button sizes, button spacing, a scroll button, color and contrast preferences, and hand preferences were implemented in the app. In the modified app, all buttons and options, wherever possible, were shown as crossing one entire page from the left edge to the right edge. More than 20 sets of color themes were implemented to meet the variety of needs of different users. In addition, in response to participant suggestions, clickable pictures were implemented for body part selection instead of word typing, and to answer questions, options were provided for users to make selections from instead of typing words being required. Multimedia contents (pictures, audios, and videos) were added into text materials as well.

**Figure 2:**
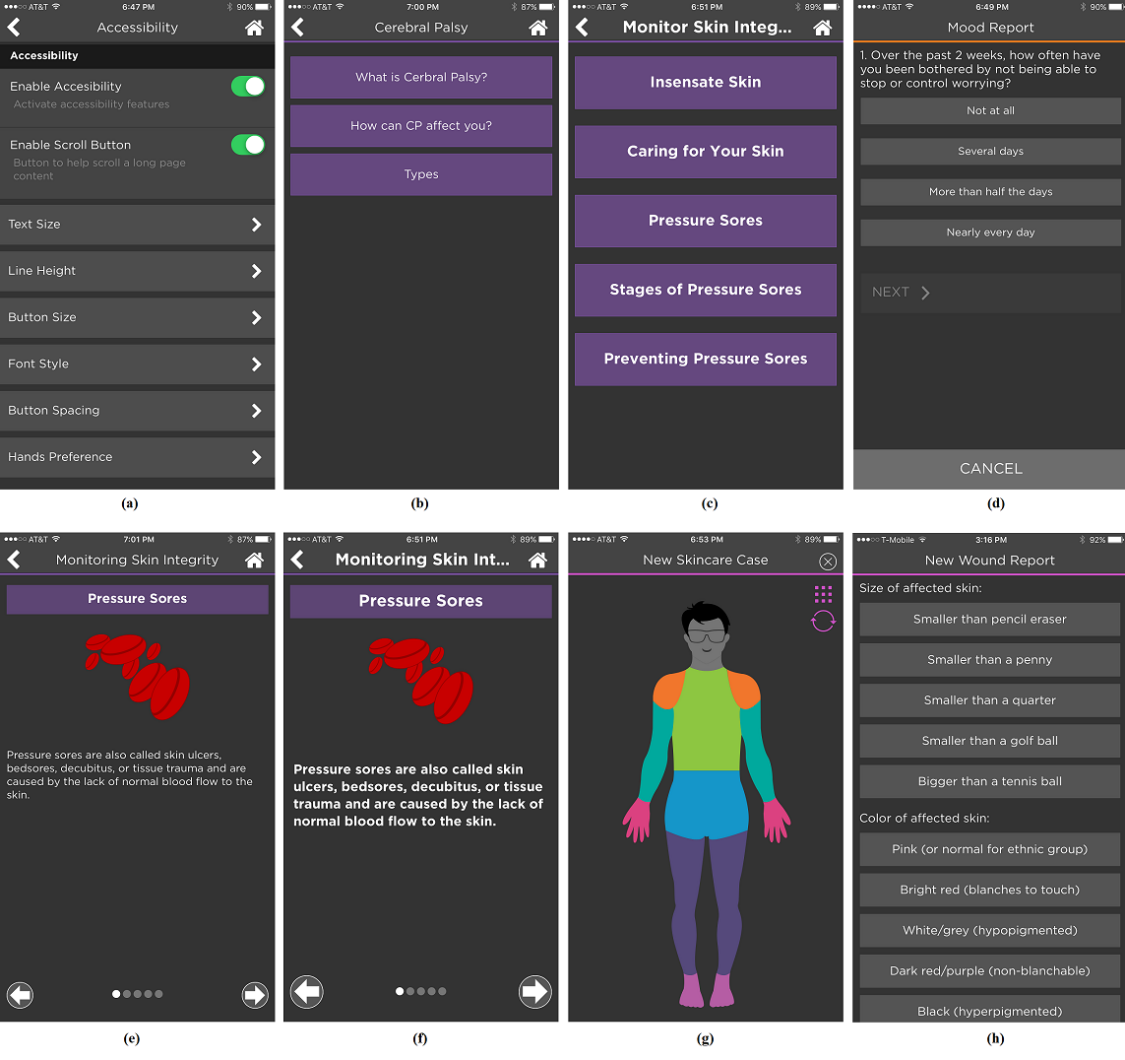
Accessibility features in the iMHere 2.0 client app. (a) List of customizable accessibility features. (b) A page in the education module without accessibility features. (c) A page in the education module with accessibility features. (d) One self-assessment question in the mood module with large button spacing. (e) A page in the education module with default settings. (f) A page in the education module with accessibility features. (g) A human body picture for body part selection in the skincare module. (h) A list of options for answering questions in the skincare module.

The first diagram in Figure 2 (top left) shows the accessibility feature settings page. The specific options for these accessibility features are listed in Table 3. The second and third diagrams in Figure 2 show the page difference in the education module before and after font size, font style, button size, and button spacing were changed. The second diagram in Figure 2 is the situation in the default setting, whereas the third diagram in Figure 2 demonstrates the situation when *large* font size, *bold* font style, *large* button size, and *large* button spacing have been applied. The fourth diagram in Figure 2 displays the situation in the mood module when only *large* button spacing has been applied. The fifth and sixth in Figure 2 are another comparison of a page in the education module before and after font size, font style, and line spacing were changed. These 2 figures also show the number of pages (using dots), current page (solid dot) in the selected section, and the left/right arrows, which can be clicked to go to the previous page or the next page, in addition to the typical page swiping.

The seventh diagram in Figure 2 shows the added human body part picture. One can simply click on the body part affected by a wound instead of using words to describe the location of wound. There are multiple pictures provided for this purpose, including the front and back of the human body (clicking the circle at the top right corner switches from front to back, not shown) and multiple pictures of a foot. One can also choose to select a body part from a list (clicking the square box icon at the top right corner to access the name list, not shown). The second, third, and fourth diagrams in Figure 2 show the edge-to-edge buttons, whereas the eighth diagram in Figure 2 shows the edge-to-edge options. The options in the eighth diagram in Figure 2 also make it possible for users to choose from among a list of answers to questions using a soft keyboard to describe the wound condition instead of having to type answers to those questions in text boxes. All figures also show the dark background and bright text.

By default, all customizable accessibility features are disabled. Once the accessibility features are enabled, users can choose the specific accessibility features according to their needs, and these features are then applied to the user interface components on the entire iMHere 2.0 client app. Users can determine whether their selections meet their own accessibility needs.

**Table 3:** Options for several accessibility features available in the iMHere 2.0 client app.

Accessibility features	Options
Font size	Small, medium^a^, large, and extra large
Font style	Normal^a^, bold
Line spacing	Narrow, medium^a^, wide, and extra wide
Button size	Small, medium^a^, large, and extra large
Button spacing	Small, medium^a^, and large
Hand preference	Left, right^a^
Scroll button	False^a^, true
Color themes	Multiple sets of color themes, such as colorful^a^, black and white, frame, and bright

^a^Default options.

### Performance on iMHere 2.0 With Accessibility Features

As mentioned earlier, all study participants in the first session of the study were able to finish the tasks given by the investigator with varying types and levels of difficulty. After the desired accessibility features were implemented into the iMHere 2.0 client app, the study participants were asked to complete the same tasks again. First, they were asked to use the app without the accessibility features (the original version of the app). Then, the study participants were given access to the accessibility features, and they made adjustments according to their needs. For instance, study participant 3 switched the hand preference from right to left because her left hand was better than her right hand, which moved all buttons listed on the right side of the screen to the left side.

Overall, when the original version of the app was used, these study participants still had the same difficulties experienced in the first session. After the accessibility features were turned on, however, not only did these study participants report that they believed that the new accessibility features were very helpful for them but their performance on the app was also consistent with their expressed beliefs. In many cases, they only needed 1 or 2 attempts to finish a task once bigger fonts and button sizes were applied instead of the several attempts required before the accessibility features were available. They could easily finish the mood self-assessment and minor skin issue reporting by clicking buttons or choosing options from either side of the mobile devices, no matter which of their hands was relatively better. For buttons that could not be changed to edge-to-edge bars, the adjustment of handedness could be used to allow the participant to move those buttons to the side easier for them to access. Therefore, it was much easier and faster for them to finish the required tasks in this study. Overall, none of the participants had any major difficulty finishing all the tasks.

All participants expressed that they were glad that they could make selections and adjustments on individual accessibility features, as this allowed them flexibility, and they felt they had some sense of control of the process.

After they finished all the tasks on the iMHere 2.0 client app with the desired accessibility features available, they orally indicated their agreement level with the 4 statements related to accessibility improvement (*These accessibility features make it easier for me to 1. click the desired buttons, 2. make selection in a list of options in the app, 3. understand the content in the app, 4. navigate different pages in the app*.). All of them chose 1 (strongly agree) in response to the 4 statements. In other words, they believed that those accessibility features made it easier for them to click buttons, make selections, navigate on pages, and read educational contents.

### Usability

As these study participants had difficulty finishing tasks on the original iMHere 2.0 client app, they were quickly tired and became frustrated. Moreover, it is known that it is difficult for some people with CP to concentrate on 1 task for a long time. As a result of these issues, we did not ask participants to fill out any type of usability questionnaire in the first session. The main purpose of the first session was to observe their performance on the app and collect their desired accessibility features.

However, after the desired accessibility features were implemented, the study participants were able to finish the given tasks (same as the ones in the first session) easily and quickly. Therefore, they were capable of answering the usability questionnaire, the SUS, as well. The calculated SUS scores from the 5 study participants were 85, 95, 92.5, 77.5, and 100. The average SUS score was 90 (SD 8.84), which means they believed the usability of the app was excellent.

Typically, if the accessibility of an app is not high (users cannot use or have significant difficulties using the app), people with disabilities do not consider the usability of the app high. However, if the usability of an app is very good according to people with disabilities, its accessibility must be high as well. Therefore, the excellent usability of the iMHere 2.0 client app indicated that the accessibility of the app was also very good.

The feedback from the study participants was positive during the informal interview at the end of the second study session. All 5 participants said that they liked the app and believed that it was easy to learn and use; they also reported that the app could be very useful for their self-management and expressed a desire to download and use the app on their own mobile device.

### Other Accessibility Features

Some accessibility features are already available in major mobile operating systems (eg, iOS and Android), for instance, VoiceOver, zoom, and bold font style. Utilizing the accessibility features offered by these mobile operating systems in a mobile app makes possible to improve the app’s accessibility. Therefore, in this study, we encouraged the study participants to evaluate some of the accessibility features offered by mobile operating systems according to their needs as well. Most of them (4/5, 80%) indicated that they were glad to know the VoiceOver feature worked fine on the iMHere 2.0 client app and said that the feature was particularly useful for reading text contents in the education module. This feature could also be used to read the text for buttons and selection options. The remaining participant expressed that the accessibility features offered by these mobile operating systems were not as flexible as the ones offered in the iMHere 2.0 app.

Some power wheelchairs offer Bluetooth connection modules, and these modules can be used to wirelessly link (pair) the control systems of these wheelchairs (eg, joystick and switch) with various types of mobile devices, such as music players, smartphones, and tablets. Once they are paired, the wheelchair user can use the joystick or switch on the wheelchair to click buttons or type words by selecting letters on the soft keyboard on the mobile device.

In this study, 3 study participants had a power wheelchair with a Bluetooth connection module installed. These wheelchairs were successfully paired with the Android tablet used in the study. The 3 study participants with Bluetooth capable wheelchairs were able to make selections on buttons and options on the app using direction and selection buttons on their wheelchairs. For some people, this offers an alternative approach to use the mobile app. For some people who could not directly operate on a mobile device because of very limited hand movement range, tremors, or another severe fine motor impairment, this could be their only way to use the app. The iPhone and iPad used in the study were not able to detect the Bluetooth signal because these power wheelchairs were missing the required module that serves as the interface between the wheelchair and Apple devices.

## Discussion

### Principal Findings

In this study, we worked with 5 study participants with different levels of dexterity impairment, collected their feedback on the accessibility of an mHealth app (iMHere 2.0 client app), implemented *customizable* accessibility features accordingly, and evaluated the mHealth app again (both accessibility and usability) to determine whether the implemented features improved the accessibility of the app and the level of usability of the updated app.

The study results indicate that before the desired accessibility features were available, these 5 study participants were able to use the app but experienced different levels of difficulty finishing some given tasks. However, after the customizable accessibility features were implemented in the app and selected by participants according to their specific needs, they were able to easily finish all the given tasks and expressed that they were highly satisfied with the updated version of the app. This indicates that our approach can be used to make mHealth apps more accessible to people with dexterity impairment.

There are multiple reasons for this satisfying outcome. First, we worked closely with these study participants to identify and analyze their needs before we designed the accessibility features for them, including taking into consideration their primary diagnosis, difficulties completing daily activities, information and communication technologies they had used in the past, mobile devices they had used in the past, and their desired accessibility features in general (including the iMHere 2.0 client app and other apps as well). The collected information was used to guide the design and implementation of accessibility features in the app. Second, we identified both the accessibility needs common among these study participants as well as the individual accessibility requests and designed those accessibility features accordingly; furthermore, we arranged these features into categories (eg, text, button, spacing, color, and handedness), which made it easy for the app users to select their desired accessibility features in the settings. Third, many of the accessibility features were implemented as *individually adjustable* components, allowing the app users to customize the user interface of the app according to their needs. This approach makes it possible to meet the needs of people with highly *diverse* types and levels of disabilities. Fourth, when we conducted the study, we took into account the device form factor (phone vs tablet) and possible uniqueness of different mobile operating systems (iOS vs Android) to make sure that the accessibility features would work well regardless of the size of mobile device or the mobile operating system.

As mentioned in the Methods section, we did not implement all the accessibility features requested by the study participants. We evaluated each requested feature first to determine the nature of the request (a need or a preference) and whether the feature could improve the accessibility of the app for this population. If the feature was only a preference, not a need, it did not have high priority on the feature implementation list. For instance, 1 participant requested very large text on buttons even though her vision was fine and she could read text contents in medium size. In this case, the request was a preference, not a need. It may be addressed in the future but not in this study. If the feature was determined to be a need, we further determined whether the feature could improve the accessibility of the app. For example, 2 study participants requested the ability to use their voice to make selections on the app because they had difficulty clicking on their desired option located in the middle of the iPad. This is a need rather than a preference. We still chose not to implement this feature in the app, however, because we believed that this particular feature would not improve the accessibility of the app and that there was a better way to make the option accessible to them (buttons crossing the page from edge to edge). We made this decision because all 5 study participants had issues with their spoken language being easily comprehensible. For 1 participant, most people who did not know her well could not understand her sentences, except for some very simple words. We actually needed her mother’s help to understand her. Moreover, 3 other participants’ spoken language was not very clear, either, including 2 who had requested this particular feature. Therefore, a natural language processing algorithm would have significant difficulties recognizing sentences generated orally by them. Most likely, the error rate of the algorithm would be very high, and hence, this feature would ultimately not improve the accessibility of the app for this group of people. This feature may be implemented in the future for a different group of people who can speak clearly but have significant difficulties using their hands to operate mobile devices.

Although these accessibility features were developed according to the needs of people with dexterity impairment, they can also be useful for other people with similar needs, such as elderly people, people with big hands, and people with visual impairment. For people who are not sensitive to color, the black and white theme (black background and white contents in most places) could be particularly useful and convenient.

### Comparison With Prior Work

There have been some studies on improving accessibility of mobile apps for people with disability [34,37] or the elderly [43]; however, most of these studies were not related to health care but focused on making it feasible for the target users to use mobile apps or touch screen mobile devices [44].

Only a small number of studies on mHealth app accessibility have been conducted, including 1 study done by Silva et al [45] and our previous studies on iMHere 1.0 for patients with dexterity impairments [29,46]. The study by Silva et al only provided a design for an accessible mHealth system. It is not clear whether the usability study that was planned with the patients was performed. In this study, we continued our tradition of user-centered development and evaluation of mHealth apps with target users of the app. In the iMHere 2.0 system, we utilized some good accessibility features identified in previous studies (eg, an adjustable app list, color-coded modules, and colored body parts) and added customizable accessibility features based on target users’ feedback to meet the needs of people with a broad spectrum of disabilities.

### Limitations

This was a small-scale qualitative study to explore the feasibility of improving accessibility of an existing app by adding customizable accessibility features into the app. This sample size may not have allowed us to identify all possible accessibility features needed by people with dexterity impairment, but the in-depth conversations with these study participants enabled us to identify several major accessibility features desired by people with dexterity impairment, which was sufficient for this study.

The study participants’ performance on the app was observed but not timed. Therefore, there is no quantitative measure of the performance difference in terms of time. This was deliberate because the major purpose of the first session was to collect information on participants’ needs, and they might have felt unnecessary pressure if they were timed. In the second session, as observed by the researchers and noted in the feedback from the study participants, it was very easy for them to finish those tasks (in seconds) after their desired accessibility features were turned on.

In the second session, study participants were asked to perform the same tasks both without and with accessibility features. Their performance when the version without accessibility features was used was the same as that in the first session. In other words, although these study participants obtained a certain level of familiarity with the app from the first session, as their difficulties were from the physical function of their hands and arms, the influence of the familiarity with the app was minimal with regard to their speed of finishing tasks.

In this study, we did not have any study participants who were color blind or had very weak vision, and therefore, the findings on color themes and contrast were not conclusive. These study participants made selections in the setting on color themes and viewed the content on the mobile app under different contrast ratios; however, they did not have significant difficulty in terms of reading the content. Therefore, to evaluate the color themes and contrast ratios, people who have color blindness and people who have weak vision should be recruited to participate in the study.

The study sessions were long (>2 hours), and the tasks were challenging (multiple attempts for a simple button click) for people with dexterity impairment when their desired accessibility features were not available. These study participants became tired and frustrated after they finished the given tasks before the accessibility features were available. Therefore, we did not ask them to complete any questionnaires in the first session. Therefore, there was no quantitative data available for comparison to show the improvement of app accessibility in the 2 sessions.

Some participants’ cognitive ability in this study was lower than normal. Therefore, although they liked the app, it was overwhelming for them to learn multiple modules in a short period. It might be better to only show them 1 or 2 modules in each session and have multiple sessions.

In this study, the study participants’ performance on the app was only observed during the study sessions. It would be beneficial to evaluate long-term use of the app and the impact of those accessibility features. A randomized clinical trial (RCT) with a group of people with disabilities, especially ones with CP, SB, SCI, is in the planning stage. The duration of the RCT will be 1 year, and the feedback from the study participants of this RCT may provide results about the app after long-term use of the app.

### Conclusions

By collecting feedback from people with disabilities and introducing customizable accessibility features, the accessibility of a mobile app can be improved. More importantly, the accessibility features added to the app in this study can be introduced in other mobile apps. This would result in the desirable outcome of making many mobile apps more accessible to people with disabilities.

## Abbreviations

CP: cerebral palsy
IRB: institutional review board
mHealth: mobile health
RCT: randomized clinical trial
SB: spina bifida
SCI: spinal cord injury
SUS: System Usability Scale
